# Health Tract, No. 181

**Published:** 1863-12

**Authors:** Mattie M. Jones


### HEALTH TRACT, No. 181.

### GRUELS AND SOUPS.

BY MRS. MATTIE M. JONES.

WHEAT-MEAL GRUEL.—Mix two tablespoonfuls of wheat-meal smoothly with a gill of cold water; stir the mixture into a quart of boiling water; boil about fifteen minutes, taking off whatever scum forms on the top. A little sugar may be added if desired.

INDIAN-MEAL GRUEL.—Stir gradually into a quart of boiling water two tablespoonfuls of Indian meal; boil it slowly twenty minutes. This is often prepared for the sick, under the name of "water-gruel." In the current cook-books, salt, sugar, and nutmeg are generally added. Nothing of the sort should be used, except sugar.

OATMEAL GRUEL.—Mix a tablespoonful of oatmeal with a little cold water; pour on the mixture a quart of boiling water, stirring it well; let it settle two or three minutes; then pour it into the pan carefully, leaving the coarser part of the meal at the bottom of the vessel; set it on the fire, and stir it till it boils; then let it boil about five minutes, and skim.

FARINA GRUEL.—Mix two tablespoonfuls of farina in a gill of water; pour very gradually on the mixture a quart of boiling water, stirring thoroughly, and boil ten minutes.

TAPIOCA GRUEL.—Wash a tablespoonful of tapioca, and soak it in a pint and a half of water twenty minutes; then boil gently, stirring frequently, till the tapioca is sufficiently cooked, and sweeten.

SAGO GRUEL.—Wash two tablespoonfuls of sago, and soak it a few minutes in half a pint of cold water; then boil a pint and a half of water, and, while boiling, stir in the farina; boil slowly till well done, and sweeten with sugar or molasses.

CURRANT GRUEL.—Add two tablespoonfuls of currants to a quart of wheat-meal or oatmeal ground, and, after boiling a few minutes, add a little sugar.

GROAT GRUEL.—Steep clean groats in water for several hours; boil them in pure soft water till quite tender and thick; then add boiling water sufficient to reduce to the consistency of gruel. Currants and sugar may also be added.

ARROW-ROOT GRUEL.—Mix an ounce of arrow-root smoothly with a little cold water; then pour on the mixture a pint of boiling water, stirring it constantly; return it into the pan, and let it boil five minutes. Season with sugar and lemon-juice.

RICE GRUEL.—Boil two ounces of good clean rice in a quart of water until the grains are quite soft; then add two tablespoonfuls of sugar, and boil two or three minutes. Currants make a good addition to this gruel.

TOMATO SOUP.—Scald and peel good ripe tomatoes; stew them one hour, and strain through a coarse sieve; stir in a very little wheaten flour to give it body, and brown sugar in the proportion of a teaspoonful to a quart of soup; then boil five minutes. This is one of the most agreeable and wholesome of the "fancy dishes." Ochre, or gumbo, is a good addition to this and many other kinds of soup.

RICE SOUP.—Boil one gill of rice in a pint of water till soft; then add a pint of milk, a teaspoonful of sugar, and simmer gently five minutes.

SPLIT PEAS SOUP.—Soak the peas all night; then cook them three or four hours, or till perfectly soft. Add a little sweet cream just before they are done.

GREEN PEAS SOUP.—Take three pints of peas, three common-sized turnips, one carrot, and the shells of the peas. Boil one quart of the largest of the peas, with the shells or the pods, till quite soft; rub through a fine colander; return the pulp into the pan, add the turnips, a carrot, sliced, and a quart of boiling water; when the vegetables are perfectly soft, add the young or smaller peas, previously boiled.

SPLIT PEAS AND BARLEY SOUP.—Take three pints of split peas, half a pint of pearl-barley, half a pound of stale bread, and one turnip, sliced. Wash the peas and barley, and steep them in fresh water at least twelve hours; place them over the fire; add the bread, turnip, and half a tablespoonful of sugar; boil till all are quite soft; rub them through a fine colander, adding gradually a quart of boiling water; return the soup into the pan, and boil ten minutes.

BARLEY SOUP.—Take four ounces of barley, two ounces of bread crumbs, and half an ounce of chopped parsley. Wash the barley, and steep it twelve hours in half a pint of water; boil slowly in a covered tin-pan five hours, and about half an hour before the dish is to be served add the parsley.

GREEN BEAN SOUP.—Take one quart of garden or kidney beans, one ounce of spinach, and one ounce of parsley. Boil the beans; skin and bruise them in a bowl till quite smooth; put them in a pan with two quarts of vegetable broth; dredge in a little flour; stir it on the fire till it boils, and put it in the spinach and parsley, (previously boiled and rubbed through a sieve.)

VEGETABLE BROTH.—This may be made with various combinations and proportions of vegetables. For example—four turnips, two carrots, one onion, and a spoonful of lentil flower. Half fill a pan with the vegetables, in pieces; nearly fill up the vessel with water; boil till all the vegetables are tender, and strain.

BARLEY BROTH.—Take four ounces of pearl-barley, two turnips, three ounces of Indian-meal, and three ounces of sweet cream. Steep the pearl-barley (after washing) twelve hours; set it on the fire in five quarts of fresh water, adding the turnips; boil gently an hour; add the cream; stir in the meal; thin it, if necessary, with more water, and simmer gently twenty minutes.

VEGETABLE SOUP.—Two good-sized turnips and Irish potatoes each; one carrot, parsnip, sweet potato, and onion each; a little parsley, chopped fine, and three tablespoonfuls of rice or pearl-barley. Slice the vegetables very thin; put them in two quarts of boiling water; let them cook three hours; then add the rice, and cook one hour longer.

